# How Technologies Assisted Science Learning at Home During the COVID-19 Pandemic

**DOI:** 10.1089/dna.2021.0497

**Published:** 2022-01-12

**Authors:** Luciano A. Abriata

**Affiliations:** ^1^ Laboratory for Biomolecular Modeling, École Polytechnique Fédérale de Lausanne and Swiss Institute of Bioinformatics, Lausanne, Switzerland.; ^2^ Protein Production and Structure Core Facility, École Polytechnique Fédérale de Lausanne, Lausanne, Switzerland.

**Keywords:** COVID-19, coronavirus, pandemic, chemistry education, biology education, do-it-yourself

## Abstract

As most other aspects of life, education was strongly affected by the lockdowns imposed to slow down the spread of the COVID-19 pandemic. Teachers at all levels of education suddenly faced the challenge of adapting their courses to online versions. This posed various problems, from the pedagogical and psychological components of having to teach and learn online to the technical problems of internet connectivity and especially of rethinking hands-on activities. The latter point was especially important for subjects who involve very practical learning, for which teachers had to find out alternative activities that the students could carry out at home. In the subjects dealing with natural sciences, impaired access to instrumentation and reagents was a major limitation, but the community turned out very resourceful. Here I demonstrate this resourcefulness for the case of undergraduate chemistry and biology courses, focusing on how do-it-yourself open technologies, smartphone-based instruments and simulations, at-home chemistry with household reagents, online video material, and introductory programming and bioinformatics, which helped to overcome these difficult times and likely even shape the future of science education.

## Introduction

**FIG. 1.**
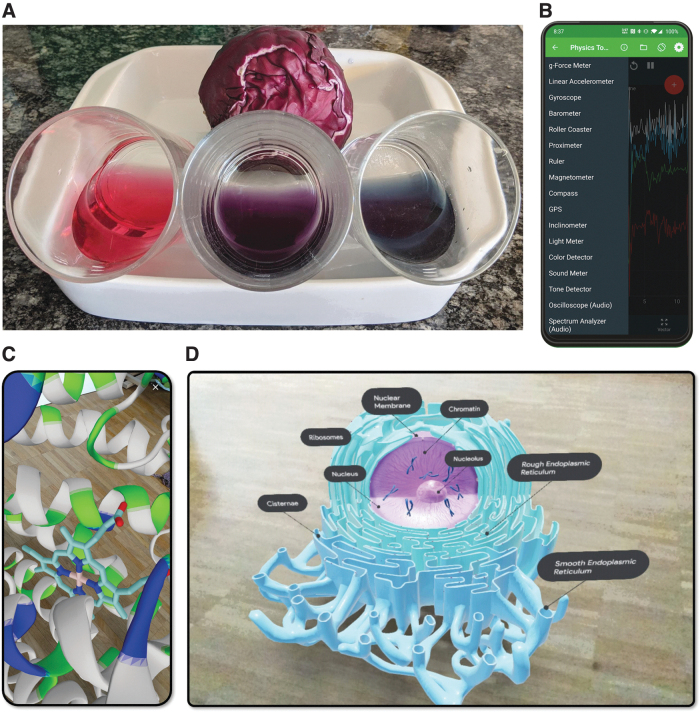
**(A)** Red cabbage-based pH indicator is popular in DIY activities for general chemistry courses. As prepared, the solution has a pH of 6–7 (center). Addition of vinegar decreases the pH to ∼4 (*left*) and addition of sodium bicarbonate increases it to ∼8 (*right*), both evidenced by clear color changes. **(B)** Screen capture of the Physics Toolbox Sensor Suite, a free smartphone app that records and logs all sensors available in a device. **(C)** Smartphone screen capture showing a heme group (sticks) inside cytochrome c oxidase (cartoons) from PDB ID 1EHK processed through moleculARweb, a website for chemistry and structural biology education at (https://molecularweb.epfl.ch). **(D)** Screen capture of a tablet showing a detailed 3D model of an eukaryotic cell nucleus of and the surrounding Golgi apparatus, directly from Google. 3D, three-dimensional; DIY, do-it-yourself.

Year 2020 saw the COVID-19 pandemic spread through the whole world striking country after country. Sooner or later, most regions of the world established lockdown protocols to control the spread rates. Among all the perturbations to our daily activities, probably one of the most important effects was on education, which got suddenly interrupted to continue only in online modality that ran for months, at all levels of education. The various facets of the impact that the COVID-19 pandemic had on education have been discussed multiple times, mostly highlighting the negative consequences on learning itself, on student and teacher psychology, on the future of global and regional economies, and on the increase of social inequalities (Anders *et al.*, 2020; Bol, 2020; Schleicher, 2020; Dietrich *et al.*, 2021; Josephson *et al.*, 2021). But the pandemic also brought opportunities to accelerate developments, rethink curricula, and push resilient educators to embrace new technologies (Dietrich *et al.*, 2020; Talanquer *et al.*, 2020; Lawrie, 2021). In fact, educators turned out very resourceful in their ways to teach their lessons online and in their ways to adapt activities to formats that students could perform at home. Here I develop specifically on one such positive point, drawn from the experiences of large number of teachers of chemistry and biology at the university level who had during the pandemic no access to laboratories yet could not simply skip practical activities for a whole year or even longer especially in the underdeveloped countries where it hit later. Around the world, a large number of educators and technologists devised activities and instrumentation that students could execute or build at home. The list is long, and spans from chemical reactions that students could perform with household compounds and monitor with do-it-yourself (DIY) instruments (Fig. 1A, B) to augmented reality (AR) applications that simulated molecular modeling kits (Fig. 1C, D) and laboratory protocols.

The use of remote teaching and learning was not new during the COVID-19 pandemic, as documented in several studies before year 2020 (Chan and Fok, 2009; Grout, 2017; Fraser *et al.*, 2019). However, the pandemic made remote education mandatory almost everywhere, thus resulting in a large burst in the number of at-home and online activities.

## DIY Heaven for Laboratory Instruments

Low-cost laboratory instruments built with relatively little expertise and a good deal of handcraft talent have flourished in the past decade (Savage, 2013; Baden *et al.*, 2015; Gibney, 2016; Ravindran, 2020), especially with the help of three-dimensional (3D) printers, open hardware policies, and increasingly easier ways to distribute information. Hackathlons, educational portals and even some dedicated programs and institutions like TReND in Africa (Trend in Africa, 2021) among one of the most visible ones, plus curious geeks, dedicated teachers and aficionados, routinely develop and release open plans for instruments such as ultraviolet (UV)-visible, infrared, Raman and fluorescence spectrophotometers, small centrifuges, chromatographic columns, low-pressure pumps, pipettes, microscopes, etc. Some require certain expertise in electronics, optics, etc. whereas others can be simply assembled from spare materials sometimes complemented with 3D-printed pieces and occasionally with elements purchased *ad hoc*. Clearly, smartphones have been the star of DIY projects in the past 5–10 years, especially as they began to include accelerometers, light and sound sensors, etc. that are exposed to programmers. Free apps that report and log all these variables in real time are abound, many even in web-based forms that do not even require installation (Abriata, 2017).

The pandemic gave place to a burst in the number of projects using such DIY instruments for experimentation, many actually employing smartphones at their core. Many teachers published their activities on social media, education-specific blogs such as ACS-promoted ChemEdXchange (ChemEdXchange website, 2021) and peer-reviewed journals such as ACS's *Journal of Chemical Education* or IUBMB's *Biochemistry and Molecular Biology Education*, reporting how they employed their activities and the student responses. During the pandemic, many of these resources built whole collections of articles compiling the most useful material for at-home work. In the next paragraphs, I comment a few cases of special interest produced during and before the pandemic, showcasing ways to cover for what students could not do at their institutions, among a very long list of other remarkable works.

Silva *et al.* (2021) introduced a webcam-based spectrophotometer that can collect emission, absorption, and fluorescence spectra with a cost <20 USD but fully equipped with a diffraction grating and a slit to select wavelengths with ∼1 nm resolution in the range from 380 to 1000 nm. Simpler colorimetric analyses without the need of light circuits to select wavelength are possible, thanks to the separate nature of channels for red, green, and blue light in camera detectors. With this technology, which needs nothing more than a webcam-equipped device such as a smartphone, Oskolok *et al.* (2021) made students determine a series of compounds in various samples. Building on a very simple protocol developed by Kuntzleman and Jacobson (2016) to experiment with Beer's law using smartphone's cameras, Madriz *et al.* (2021) instructed students how to explore the kinetics of bleaching of a food dye at their homes by monitoring color disappearance with their smartphone webcams.

Also using webcam readings to estimate absorption, Maqsood *et al.* (2021) taught their students to follow amylase-catalyzed degradation of starch and its inhibition. The amylase samples came from the students' own saliva, whereas black tea was proposed as an inhibitor. Easdon presented interesting experiments on chemical reactions and enzyme catalysis that students could perform with household reagents (Easdon, 2020). Destino *et al.* (2021) focused on enabling analytical chemistry experiments at homes too using a combination of affordable analytical volumetric flasks and pipettes with pharmacy-purchased glucose tests, pH strips, household digital scales, and smartphone-based spectrometers, plus some solution standards delivered to the students homes.

Resources such as ACS-born ChemEdXchange (ChemEdXchange website, 2021) and RSC-based Nuffield practical collection (Nuffield Practical Collection, 2021) have collected >200 practical activities including a large number of experiments to study (bio)chemical transformations with household elements.

In some developed countries, very motivated teachers could during the pandemic keep experimental activities running without recurring to household items or own-built instruments. For example, Barthet designed molecular biology laboratory activities using kits of low-cost instruments and reagents delivered to the students' homes (Barthet, 2021). It is worth noting that open-source hardware for DIY equipment can provide an intermediate solution to provide students with higher end equipment (compared with plain DIY instruments) still at low cost. For example, DIY pipettes built from 3D-printed parts, and microscopes made with phones and inexpensive lenses are quite popular.

## Commodity Web-Based AR

Inexpensive AR that runs in consumer smartphones, tablets, and laptops has exploded in the past decade, reaching very wide acceptance among the younger generations. Already before the pandemic, many educators and technologists exploited the engagement that AR evokes to build AR-based software and activities to assist teaching and learning (Bach *et al.*, 2018; Gan *et al.*, 2018; Laine, 2018; Tee *et al.*, 2018; da Silva *et al.*, 2019). It is important that even in middle-income countries a large proportion of the population has access to smartphones. This facilitates student access to the educational tools, allowing them to work at home as required during the pandemic. This is even simpler when the AR tool (same for any other kind of software) is built on the web and usable within a normal web page, because the user does not need to install nor setup any software. For example, Cortés Rodriguez *et al.* (2021) reported very wide access throughout the world to their moleculARweb website for AR education, as well as high accessibility and even indications of positive pedagogic effect on students. MoleculARweb proposes several activities that intend to make 3D visualization of chemistry and structural biology concepts easier, especially those involving dynamic aspects of matter such as variable protonation states, molecular conformations, and intermolecular interactions. moleculARweb provides a kind of AR alternative to plastic modeling kits, where by manipulating paper-printed figures students move virtual molecules, orbitals, etc. in natural 3D space. Many other smartphone apps, commercial or free, and the web-AR site BioSIM^AR^ (Fernandes *et al.*, 2021)^,^ also allow students to visualize molecules in AR, each offering certain special capabilities but all limited to static representations without dynamics or interactivity compared with moleculARweb.

AR applications to science education are not limited to molecular visualization. For example, if searched on a smartphone, Google provides WebXR experiences to observe animated annotated 3D content for a wide range of chemistry and biology subjects as exemplified in Figure 1D. Even some laboratory procedures have been replicated as AR experiences. Already before the pandemic, Tee *et al.* (2018) developed an AR system to emulate the experimental procedure for acid–base titrations. Gan *et al.* (2018) produced a system to simulate oxygen gas generation as hydrogen peroxide is oxidized by bleach, within an AR laboratory. Last, Soong *et al.* (2021) prepared a remote-controlled titration system with which students could do actual titrations at the laboratory from their homes during the pandemic, which is not exactly AR but still it is a form of altered reality.

## Social Media Videos

Online video and streaming are mostly regarded as entertainment that may in principle compete with education. However, the formidable amount of educational material on specific subjects and the number of channels dedicated to science communication, many of whose makers have become so-called influencers (“edutubers”), make a clear point for the utility of internet videos (Pattier, 2021). Video has the special power of being very engaging, descriptive, clear, and more complete than static images or spoken descriptions alone. Communicating explanations through videos is so efficient that many peer-reviewed journals currently accept movies in their SI. Let alone the *Journal of Visualized Experiments* (JoVE) that specializes on video tutorials of laboratory protocols.

Delgado *et al.* (2021) describe multiple experiences exploiting online videos from JoVE and YouTube together with laboratory sessions simulated with the commercial Labster program to carry out online cell biology laboratory sessions. Lichter showed how to use YouTube videos to teach and learn about solubility rules (Lichter, 2012). Bohloko *et al.* (2019) used YouTube videos to teach the chemistry of certain groups of elements, finding that this had a quite strong positive effect on learning by comparing with a control group. Likewise, Barry *et al.* (2016) observed that videos were more helpful to enhance learning of human anatomy. Many educators compiled online videos concerning various topics from various subjects and made them available, some even described in articles like that by Dy *et al.* (2019) focused on synthetic biology.

More broadly, the importance of social media on youth has been studied for around a decade (Van Den Beemt *et al.*, 2020). Some interesting findings include that, for example, acting out chemistry concepts in social media videos facilitates student learning (Hight *et al.*, 2021). Another important issue from the social point of view is the need for a balance between practical work and work in computers, as the latter cannot be the only source of learning (Leung and Cheng, 2021).

## An Opportunity to Develop Programming and Bioinformatics Skills

The last point I would like to highlight is how being forced to spend more time on computers opened the possibility to extend students' (and also teachers') skills on using various computer programs. Moreover, many teachers took this to larger extents, taking the chance to introduce their students to the worlds of programming and bioinformatics. Programming skills are essential today even for researchers self-perceived as “wet lab scientists.” Therefore, gaining experience through easy-to-start engaging programming languages such as Python or the combination of HTML and JavaScript (Abriata *et al.*, 2018) at a young age is a great asset for future development. Pillay introduced students to programming in Python through the Jupyter notebook, asking them to analyze various data sets (Pillay, 2020). Lorusso and Shumskaya (2020) taught their students to perform phylogenetic analyses and protein structure modeling from public SARS-CoV-2 data, effectively introducing several bioinformatic skills. Engerlberger *et al.* (2021) built a Google Collab environment with 12 tutorials that introduce students to analyzing data from phylogenetics, molecular modeling, and simulations.

## Future Perspective

The positive points of the transformation brought about by the pandemic pose many interesting ideas that will be worth exploring in the future, some of which could even become the new standard (Lockee, 2021). For example, thousands of university-level professors had to record or stream their lessons online, and they then left the corresponding videos on media such as YouTube. The next time they need to teach these lectures, wouldn't it be more efficient (and better, because some professors likely recorded certain sections multiple times to optimize communication of the content) to just refer the students back to those videos, and only after the students have watched the material then do in-person meetings to evacuate doubts and discuss specific cases, on top of the regular in-person practical activities? Likewise, instead of having 5–10 students working together on the same 20-year-old spectrophotometer, wouldn't it be far better to have much smaller groups of students utilizing their DIY instruments and recording data directly on their smartphones? The legacy of education during the pandemic, such as hybrid remote/in-person classes and more flexible deadlines for assignment completion, is being considered as the “new normal” in education. Even metrics and evaluation procedures have been adapted during pandemic times, with some changes that could possibly be adopted as the new normal (Johnson *et al.*, 2020). Perhaps laboratory sessions at home could likewise complement the hands-on experiences of students extending their regular “normal” laboratory sessions.
